# Should cities hosting mass gatherings invest in public health surveillance and planning? Reflections from a decade of mass gatherings in Sydney, Australia

**DOI:** 10.1186/1471-2458-9-324

**Published:** 2009-09-08

**Authors:** Sarah Thackway, Timothy Churches, Jan Fizzell, David Muscatello, Paul Armstrong

**Affiliations:** 1 NSW Department of Health, Locked Mail Bag 961, North Sydney NSW 2059, Australia

## Abstract

**Background:**

Mass gatherings have been defined by the World Health Organisation as "events attended by a sufficient number of people to strain the planning and response resources of a community, state or nation". This paper explores the public health response to mass gatherings in Sydney, the factors that influenced the extent of deployment of resources and the utility of planning for mass gatherings as a preparedness exercise for other health emergencies.

**Discussion:**

Not all mass gatherings of people require enhanced surveillance and additional response. The main drivers of extensive public health planning for mass gatherings reflect geographical spread, number of international visitors, event duration and political and religious considerations. In these instances, the implementation of a formal risk assessment prior to the event with ongoing daily review is important in identifying public health hazards.

Developing and utilising event-specific surveillance to provide early-warning systems that address the specific risks identified through the risk assessment process are essential. The extent to which additional resources are required will vary and depend on the current level of surveillance infrastructure.

Planning the public health response is the third step in preparing for mass gatherings. If the existing public health workforce has been regularly trained in emergency response procedures then far less effort and resources will be needed to prepare for each mass gathering event. The use of formal emergency management structures and co-location of surveillance and planning operational teams during events facilitates timely communication and action.

**Summary:**

One-off mass gathering events can provide a catalyst for innovation and engagement and result in opportunities for ongoing public health planning, training and surveillance enhancements that outlasted each event.

## Background

Mass gatherings have been defined by the World Health Organisation [1] as "events attended by a sufficient number of people to strain the planning and response resources of a community, state or nation". There is growing recognition of the public health implications of mass gatherings [1] with evidence of associated injuries [2-5], heat related illness [6,7], illicit drug-related adverse events [8], violence [9-11] and global outbreaks of infectious diseases [12,13].

For the purposes of this article the authors have focused the debate on those mass gatherings that are deemed non-emergency incidents, such as religious or sporting events, where there is usually sufficient time for planning.

One challenge facing host cities and nations is the extent to which public health practitioners should change or increase their current activities to respond to what is often a short term event with little, if any, resource enhancements.

Over the past decade, Sydney, the capital city of New South Wales (NSW), Australia, has hosted several organised mass gatherings, of both international and local importance, that meet the WHO definition. The NSW Health Department and associated Area Health Services (which together comprise "NSW Health") decided to address these challenges by enhancing public health surveillance and response capabilities for these events.

This paper explores the evolving nature of the public health response to organised mass gatherings in Sydney including the factors that influenced the deployment of resources and provides a rationale for the utility of public health planning for mass gatherings as a preparedness exercise and the building of a legacy. It is important to note that public health is only one part of an integrated whole-of-government response to preparing for mass gatherings.

## Discussion

### When does a 'mass gathering' require enhanced public health planning and response?

Sydney has an international reputation as a "party city [14]" and hosts mass gatherings of over 200,000 people at least semi-annually (including Sydney's New Year's Eve and Gay and Lesbian Mardi Gras celebrations [15]). Since 2000 the city has also hosted a number of significant international events including the Sydney Olympic and Paralympic Games in 2000 [16], the Rugby World Cup in 2003 [17], the Asia Pacific Economic Cooperation (APEC) Leaders Week in 2007, and World Youth Day in 2008 (WYD'08) [18].

Despite its "party city" reputation, only certain mass gathering events in Sydney are subject to lengthy pre-event public health planning and consequent large-scale mobilisation of public health resources.

Table 1 describes some of the features of the mass gatherings held in Sydney over the past decade where NSW Health determined that specific public health attention was required. The common characteristics of these mass gatherings were:

▪ the duration of the gathering (at least several days),

▪ the number of international visitors, and

▪ the geographical extent (with events being held in multiple locations across the city or sometimes across the whole State).

**Table 1:** A description of mass gatherings in Sydney

**Event**	**Year**	**Type of event**	**Venues/geographic spread**	**Duration of event (days)**	**Estimated age range of visitors**	**Size of gathering: Estimated numbers of visitors**		**Special Features**
							
						**International**	**Domestic**	
Summer Olympics [32]	2000	Sport	Sydney metropolitan	17	All	110,000	362,000	▪ >5 million tickets sold, ▪ 47,000 volunteers, ▪ >10,000 athletes from 199 nations, ▪ 28 sports, 300 events.
								
Paralympics [32]	2000	Sport	Sydney metropolitan	12	All	Unknown	Unknown	▪ >1 million tickets sold, ▪ 4,000 athletes from 122 nations, ▪ 18 sports.
								
Rugby World Cup [33]	2003	Sport	11 venues in 10 cities Australia-wide. Semi-finals and final in Sydney	44	All	65,000	49,948 (interstate visitors)	▪ National event ▪ 1.8 million spectators ▪ 20 teams, 48 matches. ▪ Majority of visitors from UK and Europe.
								
Asia Pacific Economic Cooperation (APEC) forum [34]	2007	Political	Sydney CBD	8 (main Sydney program)	Adult	>7,000	Unknown	▪ Included meeting of leaders of 21 member economies ▪ Low visitor numbers but high security risk.
								
World Youth Day [35]	2008	Religious	Mainly Sydney metropolitan area + pre-event activities throughout Australia	6 (main event period).	Targeted at 15-35 year-olds	110,000 registered	113,000 registered	▪ 70,000 people involved in pre-event activities in all regions of Australia. ▪ > 500,000 people attended individual events from >170 nations.

In contrast, the annual Gay and Lesbian Mardi Gras parade and New Year's Eve celebrations are short-lived events, lasting less than 48 hours. For these events the public health action focuses on health promotion activities such as safer-sex or party-safe messages and on monitoring trends in communicable disease and the detection of environmental hazards through routine syndromic surveillance [19] and mandatory notification of selected communicable diseases.

Another factor influencing the decision to enhance activities is the political or religious dimensions of some events. An international high profile has the potential to increase the risk of a terrorist attack, due to the increased global attention on one city for a short time [1]. For this reason, the APEC Leaders' Week has been included in this paper as a special type of "mass gathering": although the numerical size of the international contingent was relatively small (around 5,000) and the event was not geographically dispersed, the risk profile of participants (heads of state of 21 member economies, including the Presidents of China, Indonesia, Russia and the United States of America) increased the possibility of a terrorist attack or public disorder (as seen with G20 riots in Melbourne, 2006 [9] and London, 2009 [10] and at a number of G8 fora across the world [11]). As a result, the threats to the health of participants, summit support staff (including a large emergency services presence) and people living and working in central Sydney were considered.

Other political dimensions of mass gatherings that influence decisions to temporarily enhance public health capabilities relate to their profile and the need for governments to address community perceptions about the impact on the city and, conversely, the opportunity to maximise favourable local and international media attention.

Preparing for organised mass gatherings, however, does come at a financial and opportunity cost and the impact is highly dependent on the current level of investment in public health infrastructure and the size of the mass gathering. Additional budgets enhancements are often not forthcoming, are negligible compared to the size of the event, or insufficient to meet the expectations of event organisers or government officials. The ability for smaller jurisdictions to meet unrealistic expectations can have a negative effect on the existing workforce and could result in the de-motivation of staff and a rapid exhaustion of resources. In such situations the state of preparedness needs to be commensurate with the level of risk identified through the risk assessment and a resource prioritisation process. Preparation and response also does not need to be expensive. Many of the activities described below were achieved with only a modest budget.

### Enhanced risk assessment

In Sydney, the planning process starts with a pre-event health risk assessment, conducted up to two years before the event [11]. Importantly, initial risk assessments were periodically reviewed in the lead up to events and regular review occurred during the event as part of the daily activities of the centralised planning teams. These risk assessments are performed with input from other government agencies, such as the event organising committee, intelligence services and police - so as to co-ordinate the level of preparedness across agencies.

For each event, health risk assessments were made by an expert group using variations of a standard risk assessment methodology [1,21,22], that is: establishing the context; identifying the risks; analysing the risks; evaluating the risks and considering the likelihood of the incident occurring and the extent of its impact on human health.

Tables 2 and 3 provide a summary of the potentially significant risks identified in the pre-event period for each of the mass gatherings. Greater emphasis was placed on those risks potentially amenable to action or intervention. The assessment process included analysis of:

▪ the type of event, including duration and geographical spread of events;

▪ projected crowd densities at venues, on public transport and at accommodation sites;

▪ the characteristics of the participants, including age and expected vaccination coverage;

▪ seasonal patterns of communicable disease in NSW, Australia and in the home nations of visitors;

▪ prevalence of temporary food vending and the quality of Hazard Analysis and Critical Control Point (HACCP) plans;

▪ existing environmental hazards in the city

▪ transient environmental hazards, particularly portable or temporary toilet facilities at venues and mass accommodation sites, such as school halls, and the adequacy of hand washing facilities associated with these;

▪ access to medical facilities at venues or mass accommodation sites; and

▪ known existing security alerts.

**Table 2:** Pre-event summary risk profile of mass gatherings in Sydney: Communicable diseases

**Event**	**Year**	**Home nations of visitors**	**Main accommodation type**	**Season event held**	**New or existing communicable disease risk population and environmental risks (examples)**	**Pre-event risk level**
Summer Olympics [32]	2000	Multinational	Hotel Athletes village	Spring	▪ Late influenza season ▪ Temporary food vendors ▪ Crowding ▪ Recent local Legionella ▪ Cryptosporidium detected in the cities drinking water ▪ Circulating measles in visitor home nations	Low
Paralympics [32]	2000	Multinational	Hotel Athletes village	Spring	▪ Temporary food vendors ▪ Crowding	Low
Rugby World Cup [33]	2003	Mainly UK & Europe	Hotel	Spring		Low
Asia Pacific Economic Cooperation (APEC) forum [34]	2007	Pacific Rim	Secure hotel	Spring		Low
World Youth Day [35]	2008	Multinational, many from developing nations.	School halls/billeted/communal sleep-out at final event	Winter	▪ Influenza season ▪ Crowding	Moderate

**Table 3:** Pre-event summary risk profile of mass gatherings in Sydney: non-Communicable diseases

**Event**	**Year**	**Injury risk among visitors and spectators**	**Pre-event risk level**	**Risk of terrorist attack**	**Pre-event risk level**
Summer Olympics [32]	2000	▪ Mass gatherings at multiple sporting venues, including temporary buildings ▪ Mass gatherings and alcohol consumption at entertainment sites	Moderate	Bombing at previous Olympics in Atlanta [36]	Low-Biological Moderate-conventional
Paralympics [32]	2000	▪ Mass gatherings at single sporting venue	Low	No previous history of attack	Low
Rugby World Cup [33]	2003	▪ Multiple gatherings at various sporting venues	Low	First large, extended international mass gathering in Sydney post-11 September 2001	Low
Asia Pacific Economic Cooperation (APEC) forum [34]	2007	▪ Smaller numbers in localised area	Low	Leaders of 21 member economies Presidents of China, Russia and the United States of America	Moderate/High, biological and/or conventional
World Youth Day [35]	2008	▪ Mass gatherings at multiple venues, including temporary buildings ▪ Large pilgrimage walk through city streets	Moderate	No previous history of attack	Low

Public health planning for each of the events also built on the experience gained from previous mass gatherings and benefited from direct access to both local public health practitioners involved in those events and to the formal documentation. However, even though NSW Health has played a major role in the planning and operations of five large mass gatherings within an eight year period, knowledge transfer from previous event public health teams to the next did not always occur smoothly or completely, primarily due to changes in the personnel involved at each event. Thus, archiving of documentation and operational reports from previous events is important, as is the capture and storage, in written, searchable form, of the findings of event "post mortem" and similar debriefing activities. The inclusion of senior public health personnel with past mass gathering planning experience on relevant committees and working groups is also recommended.

The remainder of this article discusses the type of response required once the decision has been made to enhance public health planning, surveillance and response.

### Should host cities increase public health surveillance?

For all types of mass gathering, routine surveillance of communicable disease trends and environmental hazards should be maintained. However, to supplement these routine systems it is important to develop and deploy event-specific public health surveillance systems to provide earlier and/or more sensitive warning for specific risks identified through the risk assessment process. This section describes the development and refinement of special-purpose public health surveillance systems in Sydney and demonstrates the benefits to the host city in doing so.

For the Sydney Olympic Games in 2000, provisional syndromic surveillance systems were implemented in selected Emergency Departments [23], venue medical clinics [24], and cruise ships used as floating hotels for a six week period [25,26]. These surveillance systems focussed on issues of public health significance, including infectious diseases and injury. To monitor environmental and food safety hazards at Olympic venues a simple standardised reporting tool of the number and outcome of inspections was developed.

Syndromic surveillance during the Sydney Olympics was assessed by NSW Health to have been useful from a public health and political perspective. Therefore, for the Rugby World Cup in 2003, a new, highly automated Emergency Department syndromic surveillance system using routinely collected data was developed and deployed. This system continues today. Information collected includes demographic, arrival and disposition information, as well as triage notes that include important descriptive information about the nature of the presentation. Automated reports, updated every four hours, highlight unusual short-term trends in conditions of potential public health concern, including infectious disease syndromes, injury and adverse events associated with alcohol or illicit drugs [8,19]. Because this system repurposes and adds value to data which would be collected anyway, and due to its high degree of automation, it has been feasible to operate this system on a continuous basis since its inception, and to gradually expand its geographical scope. Comparative "baseline" data is essential for the successful interpretation of non-specific syndromic surveillance data.

During APEC Leaders' Week, dedicated medical clinics were opened for the delegates and their support staff. A set of syndromes of potential public health importance was developed and clinic staff were requested to notify public health personnel of any persons presenting with those syndromes. A post-event audit showed that some presentations that should have been notified to public health authorities were not, particularly 'influenza-like illness'. Thus, it is important to embed public health surveillance personnel within clinical teams in such temporary medical facilities. During APEC, the pre-existing public health near real-time Emergency Department surveillance system was also utilised to monitor event-related presentations to Sydney public hospitals. Twice daily, the ED surveillance database was searched for the keyword ("APEC") and details of these presentations were reviewed by public health staff for possible public health significance.

**Figure 1:**
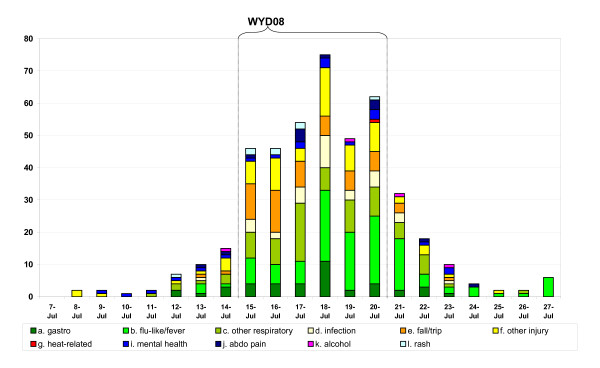
Emergency Department enhanced 'real-time' surveillance during World Youth Day 2008^# ^Sydney, using keyword searches on triage text by allocated syndrome and date of presentation*. ^#^WYD surveillance period was conducted over an extended period from 7th July 2008 till 24th July 2008. * Search terms covered the locations of the mass gatherings and relevant words such as "WYD", "WORLD", "PILG", "DIOCESE", "CROWD", "PAPAL", "POPE", and "CATHOLIC".

By the time WYD'08 was held in Sydney, the emergency department syndromic surveillance system had been operational for five years, during which time good baseline data as well as a wealth of experience in the interpretation of the often non-specific syndrome count data had been accumulated. For WYD'08, an hourly feed of ambulance emergency call data for the Sydney region was established to complement ED data feeds. The ambulance data was automatically analysed and reported. Prior to the event, ED staff were requested to flag presentations by pilgrims by entering the key words 'WYD' or 'pilgrim' in their routine free text recorded at patient triage. This provided valuable, timely and relatively pilgrim-specific syndromic information of public health importance at very little cost. Summary results of this enhanced ED surveillance are provided in Figure 1.

Many of the pilgrims attending WYD'08 were accommodated in groups of twenty to eighty, in school halls, some with limited toilet and ablution facilities, or in large, covered sporting arenas where temporary sanitation facilities were installed for the event. The Health Access Co-ordination Unit (HAC), operated by the Ambulance Service of NSW, agreed to take health-related calls from WYD'08 accommodation supervisors and to provide basic health advice or to organise dispatch of an ambulance to the accommodation, if the situation warranted. The HAC team notified the public health emergency operations centre of reports of syndromes of particular concern, such as influenza-like-illness, occurring amongst pilgrims staying in shared accommodation. These notifications led to the timely identification of a number of outbreaks of influenza^18 ^and gastroenteritis. Although it was a particularly sensitive system, like most syndromic surveillance systems, it was not especially specific, and response thresholds needed to be adjusted during the event as the resources required to investigate all potential outbreaks were depleted.

In addition, for WYD'08 NetEpi [27] an internet based public health outbreak management program was used by Public Health Units and an electronic "bedboard" system was developed and deployed at venue medical clinics. This bedboard system enabled both clinic staff and Emergency Operations Centre staff to monitor bed utilisation and waiting times in several of these temporary facilities, simultaneously and in near real-time, as well as allowing clinic staff to easily flag patient presentations of potential public health significance. Details of these flagged presentations were automatically forwarded to a separate database using the Internet. Public health personnel could then evaluate and follow-up individual cases and produce trend and other aggregate statistics.

The sophistication and effectiveness of public health surveillance has improved with each mass gathering in Sydney, building on the experience gained during previous events and benefitting from technological development and operational refinement of the state's disease surveillance infrastructure. With pre-existing, routine syndromic and notifiable communicable disease surveillance systems in place, only modest resources were required to make the necessary enhancements to ensure early warning system during mass gatherings. Because many surveillance developments used information recorded for other purposes within the NSW health system, these events have provided a tangible milestone and reason for engagement of clinical and information technology personnel who have to put aside other priorities to contribute to the development of surveillance systems.

### What level of public health response?

This section focuses on pre-event planning and training for response capabilities to potential threats to public health posed by, or heightened by, mass gatherings and the utility of these plans for ongoing public health activities.

The extent of the public health response should be determined by the outcome of the risk assessment, level of available and resources and integration of response by affected sectors.

Over time the level of coordination between response sectors to mass gatherings has strengthened in NSW. An incident control system is now fully operational that enhances communication and reduces duplication of effort between Agencies. In this system of command and control Agencies are assigned a lead role dependent on the nature of the incident or event. Strong partnerships with organising Agencies are critical and provide public health officials with access to sites and to medical centres located in venues. In the event of an emergency response during the event, the state's Disaster Plan [28] may be enacted - this plan clearly identifies the roles and responsibilities of each responding agency.

At the time of the Sydney Olympic Games, NSW had a well developed public health network across the state, comprising 17 public health units (PHU), each staffed by between ten and twenty public health practitioners, including public health physicians and nurses, environmental health officers and food inspectors. The network was supported by a central office function based in the Department of Health in Sydney. The PHU teams had extensive experience in dealing with communicable disease outbreaks and environmental hazards. At that time, however, the PHU teams had very little training in emergency management, and only limited experience in co-ordinated response across regional boundaries and with other response Agencies.

To ensure unimpeded access to and clear delineation of responsibility for Olympic Games venues, a temporary change to the *Public Health Act 1991 *(NSW) was gazetted which established a special-purpose 'Olympic Public Health Team', to which public health staff from the rural regions of NSW not affected by the Games were temporarily transferred. The size of the public health team re-deployed from rural areas was kept to a minimum to reduce any impact on routine public health services in the rural areas. The rural areas also re-focussed their work towards the most acute conditions during the period of the Games leaving a back-log of work.

Training on "Olympic-specific" response protocols and the process for implementing public health surge capacity was provided to relevant PHU personnel across the State. Also, given new surveillance systems had been established in Emergency Departments extensive training over a period of one year was provided to officers to ensure data quality and timeliness. Training can result in significant costs that include venue hire, catering, travel and salary costs for up to 60 people.

During the event a Health Olympic Coordinating Centre (HOCC) was established [20,29]. This co-located all the public health teams engaged in monitoring surveillance and response planning. Co-location was found to be an important means of keeping the teams informed of the latest intelligence and maintaining swift and reliable lines of communication. The HOCC reported directly to a whole-of-government Emergency Operations Centre lead by the Police department and also the International Olympic Committee.

The Sydney 2007 APEC event occurred in a world with heightened security concerns, post the September 11 terrorist attacks in the United States. In this environment, the need for key public health staff to hold appropriate national security clearances so as to be adequately briefed on the risk of bioterrorism or other attacks became apparent at inter-agency planning meetings.

By the time the WYD'08 event occurred most PHU teams in NSW had been trained in emergency management and had a greater level of understanding of the command, control and coordination structure described in the NSW Health disaster plan (*HealthPlan*) and in the NSW Interim Influenza Pandemic Action Plan [30]. The SARS epidemic, the establishment of highly pathogenic strains of avian influenza in animal populations in neighbouring countries, and concomitant increasing concerns about pandemic influenza had also motivated significant enhancement of resources in the field, including the establishment of a dedicated public health emergency management unit within the head office of the NSW Department of Health, and the conduct of regular field exercises, some on a substantial scale.

### Ensuring a legacy for public health

There are a number of challenges facing cities or nations which are required to respond to the public health needs of mass gathering events. Often the extent of public health preparation for a mass gathering event is driven by political rather than public health imperatives [1]. With any one-off mass gathering it is also possible that, following the event, any public health developments will not be integrated into routine practice and the valuable experience will be lost as the teams established for the events are dissolved, resulting in the loss of "corporate memory", which can be exacerbated by the failure to archive documents.

Conversely, augmentation of public health surveillance and workforce infrastructure and coordination mechanisms put in place for a mass gathering often persist after the event, and thus provide ongoing benefit and should be designed with this goal in mind. Public health functions during mass gatherings are also often provided with only modest short-term budget enhancements, however, these additional funds can sometimes be used to address both the immediate needs for the mass gathering as well as meeting longer-term requirements that would not otherwise have been addressed [31].

In the case of Sydney, the regular hosting of mass gatherings has benefited public health surveillance and response, leaving a quantifiable impact. NSW Health has been able to develop and deploy near real-time syndromic emergency department and ambulance data surveillance infrastructure on an ongoing basis, which it would not otherwise have had. Each event is now formally documented and evaluated; leaving a document trail and resources for future gatherings.

An incremental enhancement in the skills and knowledge of the NSW public health workforce has enabled improved coordination of effort, using structured and standardised mechanisms.

During the current response to the pandemic (H1N1) 2009 the previous experience of mass gatherings in Sydney has enabled NSW Health to quickly implement a formal incident control system, establish a state-wide Public Health Emergency Operations Centre within 24 hours and utilised the near-real time surveillance system to provide daily reports, seven days a week, on Emergency Department activity for influenza-like-illness. The logistics of supplying equipment such as radio transmitters, laptops, wireless internet access cards and mobile phones originally acquired for World Youth Day 2008 ensured the rapid and easy deployment of public health teams to external sites and efficient communication between sectors of government. Experience in conducting mobile influenza clinics during World Youth Day also informed the development of mobile influenza clinics during the pandemic.

In between mass gatherings, formal training programs for the public health workforce in emergency management, experience in field exercises and involvement in smaller events or events with less high profile has enhanced the workforce awareness and competence in NSW. Whilst finding adequate funding for such investments can be challenging, the long term preparedness and response benefit for future mass gatherings or large scale public health emergencies, such as pandemic influenza, could offer a large return on investment to the city, state or nation.

## Summary

▪ Not all mass gatherings of people require additional public health planning.

▪ The main drivers of enhanced public health planning for mass gatherings reflect the extent of geographical spread, number of international visitors, event duration and political and religious considerations.

▪ The extent to which additional resources are required will vary and depend on: previously acquired expertise and experience, the current level of surveillance infrastructure, workforce capacity and existing emergency management structures.

▪ One-off mass gathering events provide a catalyst for innovation and engagement and can provide an opportunity for ongoing public health planning, training and surveillance enhancements that outlast the event.

## Competing interests

The authors declare that they have no competing interests.

## Authors' contributions

ST conceived and drafted the manuscript. All authors contributed their expertise and experience to the manuscript via written and verbal communication. TC contributed to the writing of the paper and provided edits. JF wrote the sections on APEC, World Youth Day and recent bio-preparedness planning. DM provided input into all parts of the paper in particular wrote the section on PHREDSS. PA provided comments on NSW bio-preparedness response. All authors read and approved the final manuscript.

## Pre-publication history

The pre-publication history for this paper can be accessed here:


